# Genome-wide identification of foxtail millet’s TRX family and a functional analysis of *SiNRX1* in response to drought and salt stresses in transgenic *Arabidopsis*

**DOI:** 10.3389/fpls.2022.946037

**Published:** 2022-09-26

**Authors:** Shuangxing Zhang, Yang Yu, Tianqi Song, Mingfei Zhang, Nan Li, Ming Yu, Hongwei Zhou, Yanning Yang, Sihai Guo, Chunhong Xu, Yongle Tu, Jishan Xiang, Xiaoke Zhang

**Affiliations:** ^1^ College of Agronomy, Northwest A&F University, Xianyang, China; ^2^ Academy of Agricultural Sciences, Key Laboratory of Agro-Ecological Protection and Exploitation and Utilization of Animal and Plant Resources in Eastern Inner Mongolia, Chifeng University, Chifeng, China

**Keywords:** foxtail millet, thioredoxin, *SiNRX1*, drought, salt

## Abstract

Thioredoxins (TRXs) are small-molecule proteins with redox activity that play very important roles in the growth, development, and stress resistance of plants. Foxtail millet (*Setaria italica*) gradually became a model crop for stress resistance research because of its advantages such as its resistance to sterility and its small genome. To date, the thioredoxin (TRX) family has been identified in *Arabidopsis thaliana*, rice and wheat. However, studies of the TRX family in foxtail millet have not been reported, and the biological function of this family remains unclear. In this study, 35 *SiTRX* genes were identified in the whole genome of foxtail millet through bioinformatic analysis. According to phylogenetic analysis, 35 SiTRXs can be divided into 13 types. The chromosome distribution, gene structure, *cis*-elements and conserved protein motifs of 35 *SiTRXs* were characterized. Three nucleoredoxin (NRX) members were further identified by a structural analysis of TRX family members. The expression patterns of foxtail millet’s *SiNRX* members under abiotic stresses showed that they have different stress-response patterns. In addition, subcellular localization revealed that SiNRXs were localized to the nucleus, cytoplasm and membrane. Further studies demonstrated that the overexpression of *SiNRX1* enhanced *Arabidopsis’* tolerance to drought and salt stresses, resulting in a higher survival rate and better growth performance. Moreover, the expression levels of several known stress-related genes were generally higher in overexpressed lines than in the wild-type. Thus, this study provides a general picture of the TRX family in foxtail millet and lay a foundation for further research on the mechanism of the action of TRX proteins on abiotic stresses.

## Introduction

Drought, salt and temperature stresses are the main environmental factors that affect the geographical distribution of natural plants, limit the development of agriculture and endanger food security. In recent years, the frequent occurrence of extreme weather has intensified the adverse effects of abiotic stresses (Zhu, 2016). In the process of coping with external environmental pressure, plants produce various reactive oxygen species (ROS) as signaling molecules, but the excessive accumulation of ROS leads to the oxidative damage of key macromolecules in cells (Zandalinas et al., 2020). Dynamic redox balance is the basis of cell metabolism. Studies have shown that thioredoxin (TRX) can eliminate excess ROS by interacting with antioxidant enzymes to stabilize the redox equilibrium of the intracellular environment (Vieira Dos Santos and Rey, 2006; Rouhier et al., 2015).

Thioredoxins (TRXs) are the primary protein disulfide reductases found in a variety of biological cells, and they are essential for maintaining the dynamic protein redox equilibrium (Dal Piaz et al., 2010). The TRX protein was first discovered in *Escherichia coli* and has been quite conserved through evolution (Laurent et al., 1964). Its structural characteristics include a disulfide active center (CXXC). The first cysteine (Cys) can attack an oxidized mercaptan in the substrate target, leading to the formation of covalent mixed disulfide bonds between TRX and the substrate. Then, the second Cys can decompose the mixed disulfide and reduce the substrate, while the cysteine in the TRX’s active center forms a disulfide bond. Thereby, TRX is involved in many redox reactions through the reversible transformation of mercaptan–disulfide, thus enabling TRX to perform various biological functions in cells (Dal Piaz et al., 2010). Different from those in other species, plant TRXs are encoded by a large gene family, and the types of TRX are abundant in plants and located in different organelles, such as chloroplasts, mitochondria, peroxisomes, nuclei, plasma membranes and extracellular matrices, indicating that TRX family members have complex biological functions (Meyer et al., 2005; Rouhier et al., 2015). TRXs are divided into two types based on their active sites: typical TRXs (WCG/PPC active site) and atypical TRXs (XCXXC active site) (Chibani et al., 2021). The typical TRXs mainly include f, h, m, o, x, y, and z types, while the atypical TRXs mainly include CDSP32, NTRC, ACHT, APR, NRX (Nucleoredoxin), TRX-like, etc. (Michelet et al., 2013; Chibani et al., 2021). To date, the TRX family has been identified in *Arabidopsis thaliana*, rice (*Oryza sativa*) and wheat (*Triticum aestivum* L.) (Nuruzzaman et al., 2008; Nuruzzaman et al., 2012; Chibani et al., 2021; Bhurta et al., 2022). Numerous TRX types have been found to integrate gene expression and metabolic pathways, regulate various physiological processes (such as seed germination, seedling growth and flowering), and facilitate stress tolerance and defense responses to external stimuli in a wide number of studies (Apel and Hirt, 2004; Meyer et al., 2005; Richter et al., 2018; Nikkanen and Rintamäki, 2019; Jing et al., 2020; Nietzel et al., 2020; Sytykiewicz et al., 2020). These findings imply that TRXs play significant roles in plant growth and development as well as stress tolerance. However, the functions of chloroplasts and cytoplasmic TRXs in plants have been extensively studied, but the biochemical and biological functions of nuclear TRX proteins remain largely unknown.

Nucleoredoxins (NRX) are classified into the TRX family because they are densely localized in the nucleus, have two or three TRX-like domains and a C-terminal domain rich in cysteine, and have TRX-enzyme activity (Laughner et al., 1998; Marchal et al., 2014). Two *NRX* genes (*AtNRX1* and *AtNRX2*) have been identified in *Arabidopsis*, which have dual localization in the nucleus and cytoplasm. Studies have demonstrated that both the AtNRX1 and AtNRX2 proteins can reduce disulfide bonds (Marchal et al., 2014). It was demonstrated that the redox activity of GbNRX1 (*Gossypium barbadense*) was higher than that of AtTRX h, and the redox activity of three distinct TRX domains was found to be different (Li et al., 2016). In *Arabidopsis*, *AtNRX1* has been shown to regulate pollen tube growth and pollen fertility (Qin et al., 2009), and *AtNRX2* plays a physiological role in jasmonic acid (JA)-mediated trichome formation in *Arabidopsis* (Lee et al., 2020). In poplar (*Populus* spp.), *NRX1* participates in the salicylic acid (SA) signal transduction pathway (Xue et al., 2013). Studies have shown that the NRX1-mediated reduction of catalase (CAT) is an important mechanism for sustaining maximal CAT activity, and *NRX1* may be involved in directly regulating the ability of cells to detoxify hydrogen peroxide (H_2_O_2_), thus protecting plant cells from oxidative stress induced by adverse environmental conditions (Kneeshaw et al., 2017). It was found that *TaNRX1* was closely related to stress resistance in wheat (Zhang et al., 2014). The *TaNRX1* promoter was then examined further, and it was discovered that a 36 bp fragment (-193~157 bp) was the critical sequence of the *TaNRX1-D* gene in the response to osmotic stress or ABA therapy (Cheng et al., 2021). Furthermore, it was discovered that the homologous overexpression of *TaNRX1* in wheat positively regulated the drought resistance of wheat (Zhang et al., 2021). NRX has been proven to be an antioxidant protective mechanism that can regulate the status of reactive-oxygen-scavenging enzymes such as catalase and may also regulate the status of ascorbate peroxidase (APX), monodehydroascorbate reductase (MDHAR) and dehydroascorbate reductase (DHAR). These enzymes are potential targets for NRX proteins that regulate H_2_O_2_ metabolism through regulating target proteins to protect plant cells from oxidative stress (Kneeshaw et al., 2017; Calderón et al., 2018). *NRX* has significant impacts on plant growth, development and stress.

Foxtail millet (*Setaria italica*), a C_4_ crop of the Gramineae family, is widely cultivated in arid and semi-arid regions of Asia and Africa. It is known for its superior tolerance to drought, salinity and diseases, and has higher nutritional value than other major cereal crops due to its high protein and mineral content (Maharajan et al., 2021). Foxtail millet is better able to adapt to adverse agro-ecological conditions than rice, maize and wheat; requires minimal inputs; and is an indispensable plant genetic resource for agriculture and food security for poor farmers living on arid, non-cultivable and marginal lands (Neupane et al., 2022). The completion of the genome sequencing of foxtail millet in recent years has been useful for the study of the molecular biological stress resistance of foxtail millet. So far, plant TRXs have primarily focused on *Arabidopsis*, rice and wheat. A vast number of studies have indicated that TRX is vital for the maintenance of redox balance in plants, but few abiotic-stress-related studies of TRX in C_4_ foxtail millet crops have been reported. In this study, TRX family members in foxtail millet were retrieved, and their evolutionary relationships, physicochemical characteristics, subcellular localizations, gene structures, conserved motifs, promoter *cis*-elements and other parameters were predicted and analyzed. Following that, the abiotic expression patterns of *SiNRXs* under stress in the seedling stage were detected, and function analysis of *SiNRX1* was performed. We conducted this study with the purpose of providing data to enable further research into the role of the TRX family in the signaling system for the abiotic stress response in foxtail millet and improving agricultural stress resistance through genetic engineering technologies.

## Materials and methods

### Identification and chromosome distribution of the TRX family in foxtail millet

The whole-genome sequence data for foxtail millet were downloaded from the Phytozome database (https://phytozome-next.jgi.doe.gov/); then, the Hidden Markov Model (HMM) profile (ID: PF00085) with the TRX domain was downloaded from the Pfam database (http://pfam.xfam.org/). The HMMER software was used to search the genome data for foxtail millet for TRX proteins with an E-value cutoff of 1e^-3^. The candidate TRX protein sequences of foxtail millet were confirmed using the NCBI-CDD retrieval tool (https://www.ncbi.nlm.nih.gov/cdd) and SMART structure domain (http://smart.embl-heidelberg.de/smart/set_mode.cgi?NORMAL=1#).

The chromosomal location information of *SiTRXs* were obtained from the Phytozome database, and the visual analysis of the *SiTRX* chromosomes’ location was performed using the online website of MapGene2Chrom web v2 (http://mg2c.iask.in/mg2c_v2.1/).

### Protein properties and subcellular localization prediction

The amino acid number, molecular weight (MW) and isoelectric point (PI) of the SiTRX proteins were calculated using the online website ExPASy (https://web.expasy.org/protparam/). The subcellular locations of the TRX proteins were predicted using the CELLO online website (https://cello.life.nctu.edu.tw/).

### Phylogenetic analysis and classification

To analyze the phylogenetic relationship of the TRXs, a phylogenetic tree was constructed using the TRX protein sequences for foxtail millet, rice (Nuruzzaman et al., 2008) and *Arabidopsis* (Meyer et al., 2005). The protein sequence information of the family members in rice and *Arabidopsis* was downloaded from the Phytozome database (https://phytozome-next.jgi.doe.gov/). Multiple alignments of the TRX family from foxtail millet, rice and *Arabidopsis* were performed by using ClustalW with the default settings. A phylogenetic tree was constructed by using the method of maximum likelihood (ML) with 1000 bootstrap repetitions in the MEGA 7.0 software (https://www.megasoftware.net).

### Gene structure and conserved motif analysis

*SiTRX* gene sequence files (coding sequence and genome sequence) were retrieved from the Phytozome database. The gene structure was analyzed through the Gene Structure Display Server (GSDS 2.0, http://gsds.gao-lab.org/). MEME (https://meme-suite.org/meme/tools/meme) was used to analyze conserved motifs, and the maximum number parameter for a conserved base sequence was set to 10.

### Analysis of *cis*-regulatory elements

The genomic sequence (2000 bp upstream of the ATG initiation codon) of the each *SiTRX* member was obtained from the Phytozome database, and *cis*-regulatory elements were identified using the Plant CARE online website tool (http://bioinformatics.psb.ugent.be/webtools/plantcare/html/).

### Plant materials, growth conditions and treatments

The seeds of the foxtail millet variety Yugu 1 were provided by Research Fellow Jishan Xiang of Chifeng University, China, and were sown in pots and grown in a controlled incubator under controlled conditions (30°C/18°C temperature, 14 h light/10 h dark photoperiod, 60-70% humidity, 15000 lux light intensity). After reaching the three-leaf stage, the seedlings were transferred and precultured in half-strength Hoagland’s solution. When the growing seedlings reached the five-leaf stage, stress treatments were applied by transferring the seedlings to the same solution containing 20% PEG6000 and 100 mM NaCl. For cold treatment, the seedlings were transferred to a growth chamber set at 4°C. As a control, cultures were unvaryingly grown in half-strength Hoagland’s solution. Whole plants under a time series treatment (0, 1, 3, 6, 12 and 24 h; three biological replicates) were harvested, immediately frozen in liquid nitrogen and stored at -80°C for further RNA extraction.

Wild-type (WT) *Arabidopsis* is a Columbian ecotype, which was preserved by our laboratory for the genetic transformation and functional analysis of *SiNRX1*. *Arabidopsis* plants were grown both in pots with a soil mixture (vegetative soil/vermiculite = 3:1, v/v) and in a controlled incubator (25°C/20°C temperature, 16 h light/8 h dark photoperiod, 60-70% humidity, 15000 lux light intensity). All seeds were sterilized with 3% NaClO and 70% ethanol before sowing.

### Real-time quantitative PCR (qRT-PCR)

The total RNA was extracted from the leaves of the foxtail millet variety Yugu 1 using the TRIzol method, and hydroponic seedlings were tested under normal condition and different stresses (20% PEG6000, 100 mM NaCl and 4°C) for 0, 1, 3, 6, 12 and 24 h. The first-strand cDNA was synthesized by reverse transcription using the *EasyScript* ^®^ One-step gDNA Removal and cDNA Synthesis SuperMix according to the manufacturer’s instructions. qRT-PCR was carried out using a *PerfectStart* ^®^ Green qPCR SuperMix on a QuantStudio 7 Flex Real-Time PCR System following the manufacturer’s instructions. The PCR thermal profile comprised an initial denaturation at 94°C for 30 s, followed by 43 cycles of 94°C for 5 s and 60°C for 30 s. The relative expression level of each *NRX* gene (*SiTRX33* (*SiNRX1*), *SiTRX20* (*SiNRX2*) and *SiTRX25* (*SiNRX3*)) was then calculated based on the Eq. 2^−ΔΔCT^. All the samples were processed with at least three biological and three technical replicates. *SiActin* (ID: Seita.5G393000) was used as an internal control. The specific primers used above are shown in **Table S2**.

### Cloning of *SiNRXs* in foxtail millet

The cDNA was obtained as described above. Specific primers were designed using the Oligo 7 software and were used to amplify each gene (*SiNRX1*, *SiNRX2* and *SiNRX3*) fragment (**Table S2**). The PCR system used 1.0 μL of 100 ng μL^-1^ cDNA, 10 μmol L^-1^ of forward and reverse primers at 2 μL each, 2 × Phanta Max Buffer at 25.0 μL, dNTP Mix 1 μL, Phanta Max Super-Fidelity DNA Polymerase 1 μL (Vazyme, Nanjing, China), ddH_2_O 18 μL, at a total of 50.0 μL. The PCR procedure was as follows: pre degeneration at 95°C for 5 min; degeneration at 95°C for 30 s, annealing at 60°C for 30 s, extension at 72°C for 1 min 30 s, over 35 cycles; 72°C for 5 min. At a voltage of 130 V and a current of 250 mA, gel electrophoresis was used to detect whether the PCR product contained the target band. After gel recovery and purification, the PCR products were introduced into the pEASY-T_1_ cloning vector (TransGen, Beijing, China), which was used to transform DH5α competent cells (Vazyme, Nanjing, China) *via* the heat shock method. Then, positive clones were identified by PCR and sequencing.

### Subcellular localization and transcriptional activation assay

The subcellular localizations of green fluorescent protein (GFP) tags were determined for protein localization analysis. The *SiNRX1*, *SiNRX2* and *SiNRX3* coding sequences were amplified without a termination codon and fused to the Pcambia1302-GFP vector under the control of the CaMV35S promoter (for the primers, see **Table S2**). The GFP fusion vector was transformed into tobacco epidermal cells, and the GFP fluorescence was observed using a confocal laser scanning microscope (Olympus, Japan) 48 hours later. The empty vector pGBKT7 and recombinant vector pGBKT7-*SiNRX* were transferred into a yeast two-hybrid (Y2H) gold strain (for the primers, see **Table S2**), and cultured upside down in SD/-Trp medium at 30°C. The colony growth was observed 2-3 days later. Then, monoclonal colonies were cultured in SD/-Trp medium, and 1 μL was extracted and inoculated on SD/-Trp and SD/-Trp/-His/-Ade media, respectively. At a temperature of 30°C, the colony growth was observed and recorded after the inverted culture for 3-5 days.

### Generation of transgenic *Arabidopsis* plants and phenotypic analysis

For *Arabidopsis* transformation, the ORF of *SiNRX1* was cloned into the pBI121 vector at the *BamHI* and *SacI* sites in a manner that created a pBI121–*SiNRX1* construct under the control of the CaMV35S promoter (for the primers, see **Table S2**). The transformation of *Arabidopsis* was performed using the *Agrobacterium*-mediated transformation method (Clough and Bent, 1998). *Agrobacterium tumefaciens* inflorescence of *Arabidopsis* was immersed in a transformation medium containing *Agrobacterium* for 1 min. With the help of transformation media, *Agrobacterium tumefaciens* can penetrate the plant cell wall and plasma membrane, infect the female gametophyte, or embryo sac, and integrate the T-DNA fragments with the target genes and components into the genome of the female gametophyte (Zhang et al., 2006). After infection with WT *Arabidopsis* (T_0_), seeds collected from the T_0_ generation were considered T_1_ generation seeds (mixed collection). The T_1_ generation seeds were screened on Murashige and Skoog (MS) medium containing 50 mg L^-1^ kanamycin, and the transgenic T_1_-generation positive seedlings were directly screened out and the T_2_ generation seeds were harvested (single plant). T_2_ generation seeds were screened on MS medium containing 50 mg L^-1^ kanamycin. If the ratio of positive to negative seedlings met the separation ratio of 3:1, positive seedlings were transplanted and T_3_ seeds were collected (single plant). T_3_ seeds were screened on MS medium containing 50 mg L^-1^ kanamycin, transplanting and culture were carried out, and transgenic homozygous lines were finally obtained. Three independent homozygous T_3_ seedling lines were chosen for subsequent experiments.

For the growth assay, 7-day-old WT and transgenic *Arabidopsis* seedlings were grown on MS medium and then transferred to pots filled with soil and vermiculite (3:1, v/v) for an additional 2 weeks. By counting the days since the last watering, the drought-stress treatment was performed for 20 days, and rehydration was performed for 3 days. Ultimately, the number of surviving plants was recorded, and the survival rates were determined. Each experiment (including approximately 36 plants) was repeated three times. For the salt-stress treatment, three-week-old *Arabidopsis* were irrigated with 150 mM NaCl every 5 days and irrigated 3 times for 15 days (3 irrigations in total). Then, the number of surviving plants was recorded, and the survival rates were counted. Each experiment (including approximately 36 plants) was repeated three times. For the water-loss-rate assay, when the seedlings had grown to three-week-old, the *in vitro* water loss rate (WLR) of the WT and the overexpression (OE) plants of the same size and growth rate were measured. The weights (W_2_) of the plants at 0, 0.5, 1, 2, 3 and 5 h were measured under constant conditions, where 0 h was determined as the fresh weight (W_1_), and the calculation formula was WLR= (W_2_-W_1_)/W_1_×100% (Mao et al., 2021).

### Measurement of the MDA, proline content, antioxidant enzyme activity and transcriptional profiles of stress-related and antioxidant-related genes

To analyze physiological and biochemical indexes under drought stress, the three-week-old *Arabidopsis* seedlings were treated with normal and water withholding for 15 days. To analyze physiological and biochemical indexes under salt stress, the three-week-old *Arabidopsis* seedlings were kept in normal conditions and treated with 150 mM NaCl for 10 days. The leaves were harvested to determine the MDA content, as well as the proline content (Li et al., 2020). The antioxidant enzymes were extracted, and the activities of cellular CAT, POD and SOD were determined according to the instructions of the antioxidant enzyme assay kits (Solarbio, cat nos. BC0200, BC0090 and BC0170). qRT-PCR was used to analyze the expression levels of stress-related and antioxidant-related genes under drought and salt stresses, including *AtP5CS1*, *AtRD29A*, *AtDREB2A*, *AtCAT1*, *AtPOD1* and *AtSOD* (*Cu/Zn*). In addition, qRT-PCR was used to analyze whether the overexpression of *SiNRX1* in *Arabidopsis* affected the expression level of *AtNRXs* in *Arabidopsis*. *Tubulin beta-2* (ID: AT5G62690) was used as an internal control (for the primers, see **Table S2**).

### Statistical analysis

The statistical analysis was performed using Excel (version 2016) and SPSS (version 2020). The mean values were statistically compared using Student’s t-test (* indicates *p* < 0.05, and ** indicates *p* < 0.01).

## Results

### Identification of 35 *TRX* genes in the foxtail millet genome

The NCBI-CDD retrieval tool and a SMART domain search were used to select protein sequences with complete TRX or TRX-like domains. Thereby, 35 candidate *SiTRX* genes were identified in the foxtail millet genome. The *SiTRX* genes were mapped to all nine foxtail millet chromosomes and were named according to the distribution of chromosomes (**Figure S1**). Among all the chromosomes, the *SiTRX* genes were unevenly distributed across the genome. Seven *SiTRX* genes were found on chromosome 9, while five *SiTRX* genes were found on each of chromosomes 2, 3 and 7, and at least one gene was found on each of chromosomes 6 and 8.

### Analysis of the physicochemical properties and subcellular localization prediction of the TRX family

The TRX proteins of foxtail millet range in size between 100 and 580 amino acids. The relative molecular weights (MWs) vary from 11,086.94 to 64,177.87 Da, and about half (51.43%) have low isoelectric points (PIs) (PI < 7.0) (**Table S1**). Through the prediction of the subcellular localization of the TRX family, TRX members may be distributed in different organelles, such as chloroplasts, cytoplasm, nuclei, mitochondria or plasma membranes. These details indicate that TRX members may play roles in different parts of cells. Therefore, TRX members presumably play roles in different aspects of plant growth and development.

### Phylogenetic analysis of the TRX family

**Figure 1:**
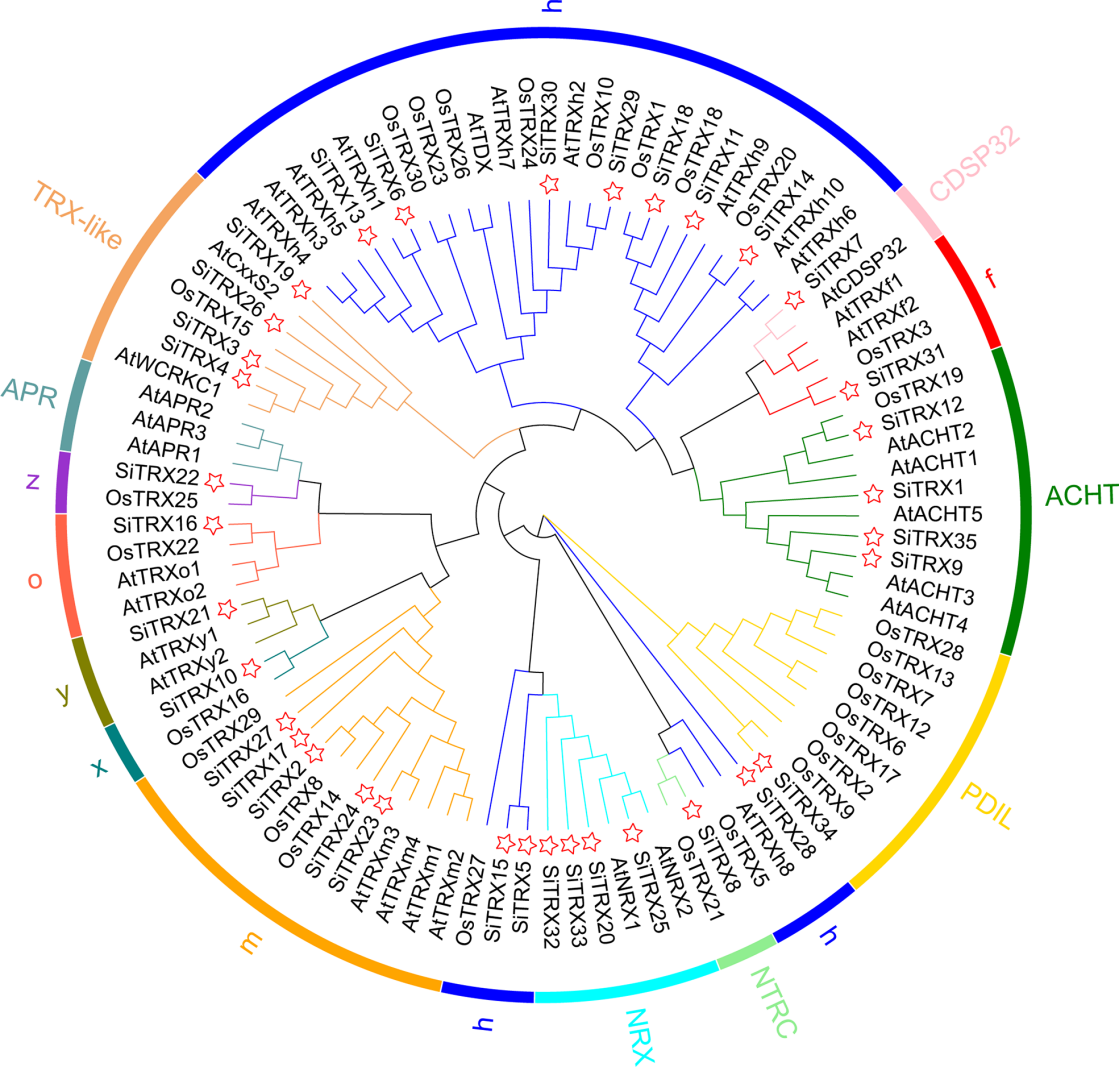
Phylogenetic relationships of TRX family proteins in foxtail millet, *Arabidopsis* and rice. The phylogenetic tree was constructed using the maximum likelihood method in the MEGA 7.0 software. The red star represents SiTRX. The species’ acronyms are shown before each TRX-protein name: Si, *Setaria italica*; At, *Arabidopsis thaliana*; Os, *Oryza sativa*.

According to the evolutionary relationships and BLAST search against the NCBI database (https://blast.ncbi.nlm.nih.gov/Blast), the TRX proteins were divided into h, o, y, z, x, m, f, ACHT, TRX-like, APR, NRX, NTRC, PDIL and CDSP32 types (**Figure 1**). Type h has the most members, including ten SiTRXs, eleven AtTRXs and ten OsTRXs. This is followed by type m, which includes five SiTRX members, four AtTRX members and three OsTRX members. Type f includes one SiTRX member, two AtTRX members and one OsTRX member. Type z includes one SiTRX member and one OsTRX member. Type o includes one SiTRX member, two AtTRX members and one OsTRX member. Type y includes one SiTRX member and two AtTRX members. Type x includes one SiTRX member and one OsTRX member. ACHT includes four SiTRXs, five AtTRXs and one OsTRX. TRX-like includes four SiTRXs, two AtTRXs and one OsTRX. APR includes three AtTRXs. NRX includes four SiTRXs and two AtTRXs. NTRC includes one SiTRX and one OsTRX. PDIL includes one SiTRX and eight OsTRXs. However, none of the SiTRXs in foxtail millet are classified as the APR type. Type h is divided into three branches, indicating different evolutionary origins. Overall, the results show that the relationship between TRX family proteins in foxtail millet and rice was closer than that in *Arabidopsis*, which is in line with the evolutionary relationships of the plants.

### Gene structure and conserved motif analysis of the SiTRX family

**Figure 2:**
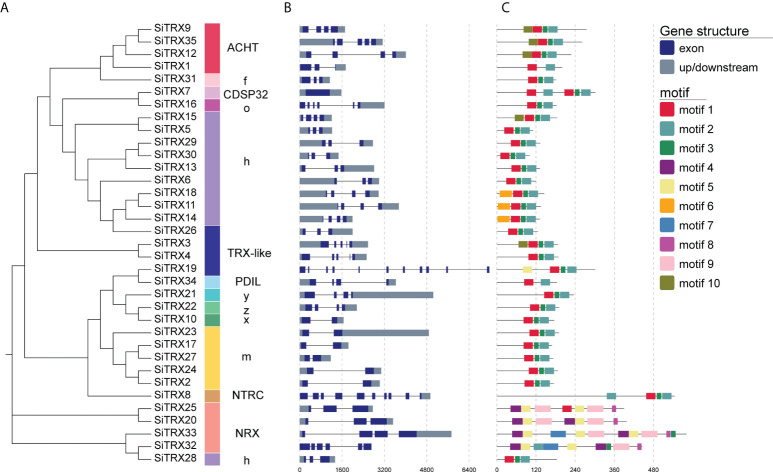
Evolutionary relationship, gene structure and conserved motif analysis of SiTRX members. **(A)** The evolutionary relationship of TRX in foxtail millet. **(B)** Gene structures of *SiTRXs*. The intron–exon structures were obtained by comparing the coding and genomic sequences using the GSDS website. **(C)** Motif composition, with the ten conserved motifs indicated with boxes of different colors.

On the basis of the phylogeny and gene structure, the 35 SiTRX members were divided into multiple branches, including h, o, y, z, x, m, f, ACHT, TRX-like, NRX, NTRC, PDIL and CDSP32 types, which were consistent with the phylogenetic relationship (**Figure 2A**). Exon/intron organization analysis further revealed that most of the *SiTRXs* contained two to five exons, with similar gene structures for each type (**Figure 2B**). The gene lengths greatly differed among the members of the *TRXs*, indicating that great variation in the exon/intron lengths of this family of genes occurred during evolution, which may have led to diversity in function.

In addition, whether SiTRX proteins had a conserved domain was analyzed (**Figure S2**). *SiTRXs* encode proteins with conserved TRX or TRX-like domains. In addition to the TRX-like domain, SiTRX20, SiTRX25 and SiTRX33 have a C-terminal domain rich in cysteine, and SiTRX8 contains another redox domain. To better understand the structural characteristics of SiTRX proteins and provide clues regarding their functions, the conserved motifs of SiTRX proteins were analyzed (**Figure 2C**). All of motif 1 and motif 2 are present in typical TRXs. The motif found in atypical TRXs differs from the conserved motifs observed in typical TRXs. Interestingly, conserved motifs of NRX are included in motif 4, motif 5, motif 8 and motif 9, which are different from the other types of conserved motifs in TRX. The functions of most of these conserved motifs remain to be elucidated.

### *Cis*-regulatory elements of *SiTRX* genes

A further analysis using Plant CARE revealed *cis*-regulatory elements in the promoters of the *SiTRX* genes, and these elements were related to different functions (**Figure S3**). In addition to the basic elements (CAAT-box and TATA-box), a large number of light-responsive, environmental-stress-related, hormone-responsive, development-related, site-binding-related and other *cis*-regulatory elements were identified in 35 *SiTRX* genes in the promoter region. Most of the *SiTRX* genes had light-response elements (G-box, ACE, GT1-motif, GATA-motif, Box 4 and TCT-motif). About 54% of the *SiTRX* genes contained low-temperature-response elements (LTR), 57% of the *SiTRX* genes contained drought-induction-response elements (MBS), 82% of the *SiTRX* genes contained antioxidant-stress-response elements (ARE), and 48% of the *SiTRX* genes contained hypoxia-specific-induction elements (GC-motif). About 88% of the *SiTRX* genes contained hormone-response elements and methyl-jasmonate response elements (CGTCA-motif and TGACG-motif), about 82% of the *SiTRX* genes contained abscisic-acid-response elements (ABRE, ABRE4, and ABRE 3A), and about 45% of the *SiTRX* genes contained gibberellin-response elements (P-box and GARE-motif). In addition, some genes contained elements related to growth and development, such as meristem expression (CAT-box), the material metabolism regulatory element (O_2_-site), MYB (MER), 45% of the MYBHv1 binding site (CCAAT-box), etc. These results indicate that *SiTRX* genes may be regulated by multiple *cis*-acting elements and play important roles in plant photosynthesis, growth and development, hormone regulation and the stress response.

### Expression analysis of *SiNRX* genes in response to drought and salt stresses

**Figure 3:**
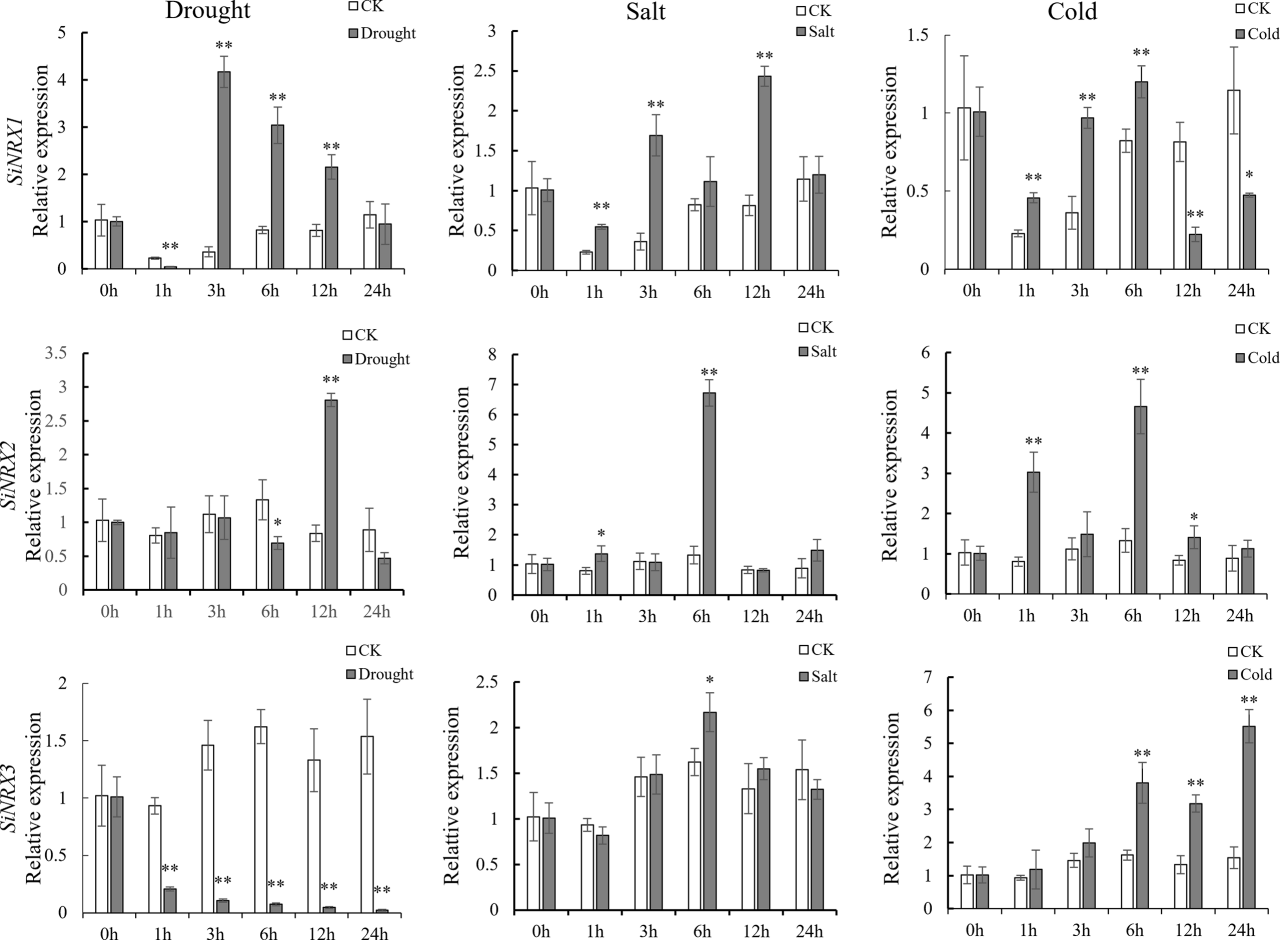
Expression patterns of *SiNRX1*, *SiNRX2*, *SiNRX3* under abiotic stresses. qRT-PCR analysis of *SiNRX1*, *SiNRX2*, and *SiNRX3* in foxtail millet leaves in response to diverse treatments (drought [20% PEG6000), salt (100 mM NaCl), and cold (4°C)]. CK as control (without stress treatment). Standard deviations are indicated by error bars (mean ± SD and n = 3). Each time point was compared to the control group, and student’s t-test was used to calculate significance: *indicates *p* < 0.05, and **indicates *p* < 0.01.

NRX is the first TRX found to be located in the nucleus, and its specific biological function remains to be further explored. In order to verify the results of the *cis*-element analysis and further reveal the responses of *SiNRX* genes (*SiTRX33* (renamed *SiNRX1*)*, SiTRX20* (renamed *SiNRX2*) and *SiTRX25* (renamed *SiNRX3*)) to different stresses, qRT-PCR was used to detect the expression of *SiNRX* genes in foxtail millet leaves treated with normal condition, 20% PEG6000, 100 mM NaCl and 4°C (**Figure 3**). Under drought treatment, compared with the normal condition, the expression level of *SiNRX1* decreased rapidly at 1 h, then increased and reached the highest level at 3 h; the expression of *SiNRX2* decreased at 6 h, then increased and reached the highest level at 12 h, and the expression level began to decline and returned to normal at 24 h; the expression of *SiNRX3* decreased dramatically at 24 h. Compared with that of the other two genes, the expression of *SiNRX1* increased the most under drought stress. During salt treatment, relative to the normal condition, the expression level of *SiNRX1* gradually increased from 1 h to 12 h and reached the highest level; *SiNRX2* and *SiNRX3* reached their highest expression level at 6 h. On the whole, the mRNAs of *SiNRX1*, *SiNRX2* and *SiNRX3* showed a trend of first reaching their highest levels and then declining. Under a low-temperature treatment at 4°C, compared with the normal condition, the level of *SiNRX1* showed a trend of first increasing and then decreasing; *SiNRX2* reached its highest level at 6 h, *SiNRX3*;s mRNA level increased gradually over 24 h. Our results clearly show that different *SiNRX* genes respond differently to different abiotic stresses in foxtail millet leaves.

### Subcellular localization of the SiNRXs and the transcriptional activation assay

**Figure 4:**
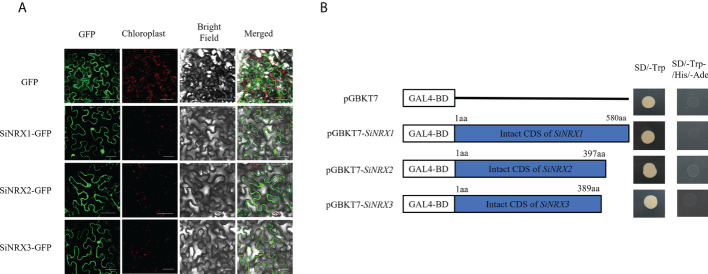
Subcellular localization and transcriptional activation of SiNRX1, SiNRX2 and SiNRX3. **(A)** Recombinant plasmid and control plasmid were transiently expressed in tobacco cells. The scale bar represents 50 μm. **(B)** The ORF fragments of *SiNRX* genes were ligated with pGBKT7 to construct a fusion vector. The yeast cells harboring SiNRX fusion vectors were cultured on the SD/-Trp and SD/-Trp/-His/-Ade.

NRX, named nucleoredoxin because it was originally found in the nucleus, was predicted to be localized in the cytoplasm, so subcellular localization was used to verify this prediction. As a control, the fluorescence signal from a GFP–null fusion protein was measured in the nucleus, cytoplasm and membrane, and the fluorescence signal from a GFP–SiNRX fusion protein was measured in the same manner as that for the control. The fusion proteins SiNRX1–GFP, SiNRX2–GFP and SiNRX3–GFP showed green fluorescence signals on the nucleus, cytoplasm and membrane (**Figure 4A**). The subcellular localization analysis indicated that SiNRX1, SiNRX2 and SiNRX3 were all localized in the nucleus, cytoplasm and membrane. The experimental results are reasonably consistent with the predictions. Yeast transfected with pGBKT7 and the recombinant vector pGBKT7–*SiNRX* could grow in SD/-Trp medium with no significant difference in growth rate. The yeast could not grow on SD/-Trp/-His/-Ade medium, indicating that SiNRX had no self-activation effect (**Figure 4B**).

### Overexpression of *SiNRX1* gene enhanced the drought resistance and salt tolerance of transgenic *Arabidopsis*

**Figure 5:**
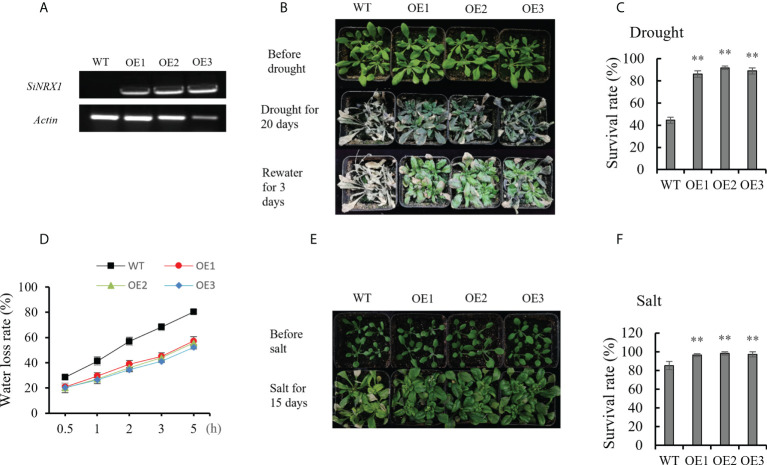
The phenotype analysis of OE lines and WT under drought and salt stresses. **(A)** Gene verification of *SiNRX1* in OE lines by PCR. **(B)** The phenotype of OE lines after drought treatment for 20 days. **(C)** The survival rate of OE lines and WT after water withholding for 20 days. **(D)** The water loss rate of leaves *in vitro* measured at 25°C. **(E)** The phenotype of OE lines and WT after salt treatment (150 mM NaCl) for 15 days. **(F)** The survival rate of OE lines and WT after salt treatment for 15 days. OE: *SiNRX1* transgenic *Arabidopsis* lines; WT: wild-type *Arabidopsis* lines. Standard deviations are indicated by error bars (mean ± SD and n = 3). Student’s t-test was used to calculate significance: ** indicates p > 0.01.

From the perspective of the structural domains of different NRX members, compared with the other two types, NRX1 has three TRX-like structural domains, so it was presumed that its redox activity would be higher than that of the other two types. According to the qRT-PCR, *SiNRX1* was more highly expressed under different stresses. Consequently, *SiNRX1* was taken as the research object for further functional analysis. To analyze the function of *SiNRX1* in drought resistance and salt tolerance, *SiNRX1-*transgenic *Arabidopsis* plants were generated under the control of the CaMV35S promoter. After RT-PCR, no bands were detected in wild-type (WT) lines, while bands were detected in overexpression (OE) lines, indicating the successful transformation of *SiNRX1* in *Arabidopsis* (**Figure 5A**; **Table S2**). Then, three independent T_3_ lines with homozygous *SiNRX1* overexpression (OE1, OE2 and OE3) were selected for further functional analyses. WT and transgenic seedlings were grown for 3 weeks under normal conditions. After 20 days of drought treatment, WT plants were severely withered or even bleached, while OE lines suffered less damage than WT. After 3 days of rehydration treatment, it was found that the new leaves of OE lines recovered better than those of the WT (**Figure 5B**). The survival rates of the three OE lines were 86.11%, 91.66% and 88.88%, respectively, while the survival rate of the WT lines was 44%. Moreover, the survival rates of the OE lines were about 42% higher than those of the WT (**Figure 5C**). WT and transgenic seedlings at three weeks old were each treated with 150 mM NaCl for 15 days. OE lines had deeper leaves, while WT lines had wilted or even white leaves (**Figure 5E**). The survival rates of the OE lines (96.29%, 98.14%, 97.22%) were higher than those of the WT (85.19%) (**Figure 5F**). In general, *SiNRX1* overexpression improves the survival rate of *Arabidopsis* plants under drought and salt stresses. The analysis of the water-loss rate at 5 h showed that the *in vitro* water-loss rate of the leaves of the WT was 80.33%, and that of the OE lines was 55.06%, on average. The water-loss rate of the WT lines was about 25% higher than that of the OE lines (**Figure 5D**).

**Figure 6:**
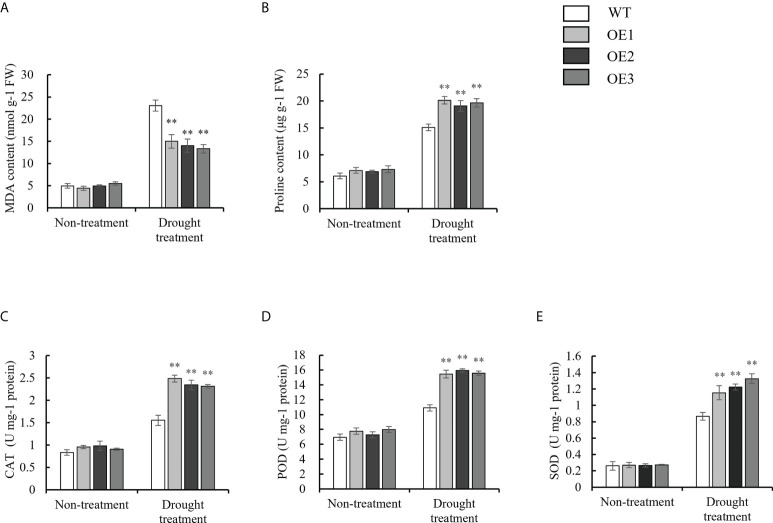
Evaluation of physiological indices of OE lines and WT responsive to drought stress. **(A)** MDA content. **(B)** Proline content. **(C-E)** Alterations of Activities of CAT, POD, and SOD. Standard deviations are indicated by error bars (mean ± SD and n = 3). Student’s t-test was used to calculate significance: ** indicates p > 0.01.

**Figure 7:**
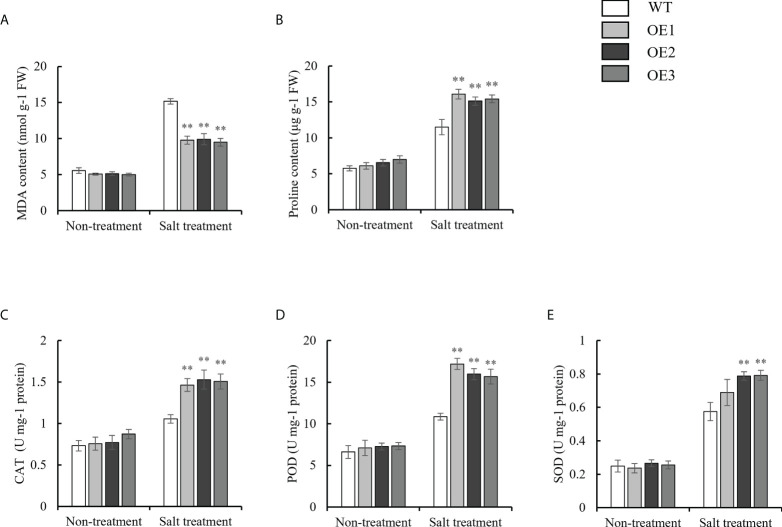
Evaluation of physiological indices of OE lines and WT responsive to salt stress. **(A)** MDA content. **(B)** Proline content. **(C-E)** Alterations of Activities of CAT, POD, and SOD. Standard deviations are indicated by error bars (mean ± SD and n = 3). Student’s t-test was used to calculate significance: ** indicates p > 0.01.

To investigate the physiological changes in the OE and WT lines, the malondialdehyde (MDA) content; proline content; and catalase (CAT), peroxidase (POD) and superoxide dismutase (SOD) activity were each measured under normal growth conditions, as well as drought and salt stresses. Under normal conditions, there were no significant differences in the MDA content; proline content; and CAT, POD and SOD activity between the WT and OE lines. Under drought stress, the MDA contents of the OE lines were 15.00 nmol g^-1^ FW, 14.01 nmol g^-1^ FW, and 13.31 nmol g^-1^ FW, respectively, while the MDA contents of the WT were 23.03 nmol g^-1^ FW (**Figure 6A**). Finally, the MDA content in the OE lines and WT was significantly upregulated under drought stress, but the increased MDA content in the OE lines was significantly lower than that in the WT. However, the trend of the proline content was opposite to that for the MDA content under drought stress (**Figure 6B**). For the antioxidant enzyme system (CAT, POD and SOD), the CAT activity of the OE lines was 2.48 U mg^-1^ protein, 2.34 U mg^-1^ protein, and 2.31 U mg^-1^ protein, respectively. On the other hand, the CAT activity of the WT was 1.55 U mg^-1^ protein under drought stress (**Figure 6C**). The trend of the POD and SOD activity was consistent with that for the CAT activity under drought stress (**Figures 6D**, **E**). Under salt stress, the MDA contents of the OE lines (9.77 nmol g^-1^ FW, 9.90 nmol g^-1^ FW, 9.48 nmol g^-1^ FW) were lower than those of the WT plants (15.16 nmol g^-1^ FW) (**Figure 7A**). However, The proline content and antioxidant enzyme (CAT, POD, SOD) activity of OE lines were higher than WT under salt stress (**Figures 7B**–E). In a word, under drought and salt stresses, the activities of CAT, POD and SOD in the OE lines were higher than those of the WT. These results indicate that the antioxidant capacity and ROS-scavenging capacity of the OE lines increased compared to those of the WT under drought and salt stresses, which resulted in the observed enhanced drought resistance and salt tolerance of the transgenic lines.

### *SiNRX1* expression affects the transcriptional levels of stress-related and antioxidant-related genes in *Arabidopsis*

**Figure 8:**
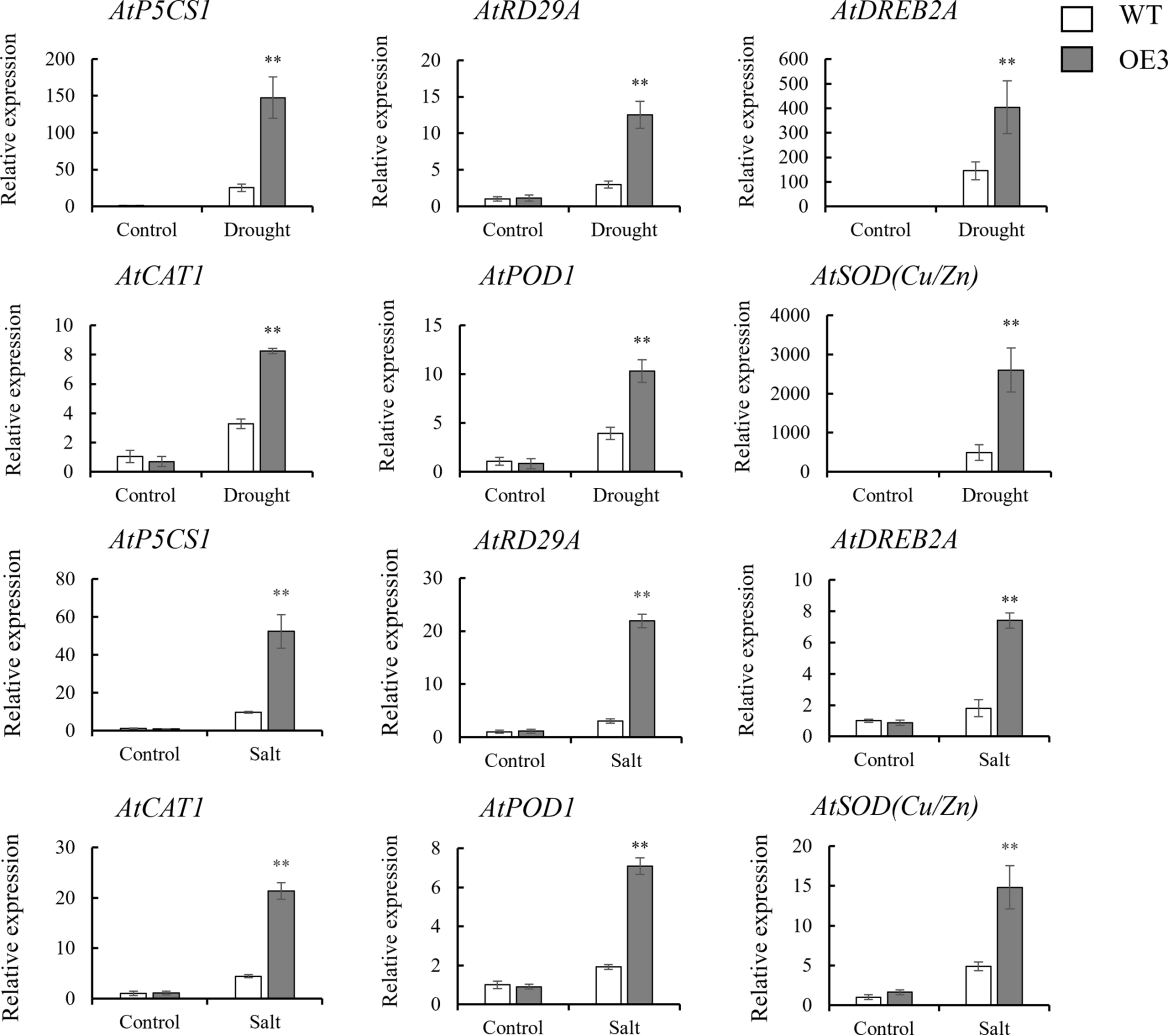
The expression levels of stress-related genes in the OE3 line and WT under stresses. stress-related genes—*AtP5CS1*, *AtRD29A* and *AtDREB2A*; antioxidant-related genes —*AtCAT1*, *AtPOD1* and *AtSOD* (*Cu/Zn*). Stresses include drought stress and salt stress. Standard deviations are indicated by error bars (mean ± SD and n = 3). Student’s t-test was used to calculate significance: ** indicates p > 0.01.

In order to further analyze the mechanism of the response of *SiNRX1* genes to drought and salt stresses, several stress-related genes and antioxidant-related genes were chosen for observation at the transcriptional level. Three stress-related genes (*AtP5CS1*, *AtRD29A* and *AtDREB2A*) and three antioxidant-related genes (*AtCAT1*, *AtPOD1* and *AtSOD* (*Cu/Zn*)) were selected for the gene expression analysis of the WT and OE lines after drought and salt stresses (**Figure 8**). Under normal conditions, there was no significant difference in the expression levels of the six genes (*AtP5CS1*, *AtRD29A*, *AtDREB2A*, *AtCAT1*, *AtPOD1* and *AtSOD* (*Cu/Zn*)). Under drought stress, the expression level of *AtP5CS1* in the OE3 line was 5.8-fold higher than WT; the expression level of *AtRD29A* in the OE3 line was 4.1-fold higher than WT; the expression level of *AtDREB2A* in the OE3 line was 5-fold higher than WT; the expression level of *AtCAT1* in the OE3 line was 2.5-fold higher than WT; the expression level of *AtPOD1* in the OE3 the line was 2.6-fold higher than WT; the expression level of *AtSOD* (*Cu/Zn*) in the OE3 the line was 5.3-fold higher than WT. Under salt stress, the expression level of *AtP5CS1* in the OE3 line was 5.1-fold higher than WT; the expression level of *AtRD29A* in OE3 the line was 7.2-fold higher than WT; the expression level of *AtDREB2A* in the OE3 line was 4.1-fold higher than WT; the expression level of *AtCAT1* in the OE3 line was 4.8-fold higher than WT; the expression level of *AtPOD1* in the OE3 line was 3.6-fold higher than WT; and the expression level of *AtSOD* (*Cu/Zn*) in the OE3 line was 3-fold higher than WT. In general, the expression levels of six genes in the OE3 line were significantly upregulated under drought and salt stresses, although the levels of the upregulation of each gene were different under different stresses. The results indicate that the heterologous expression of *SiNRX1* may enhance the drought and salt resistance/tolerance of *Arabidopsis* by enhancing the expression of stress-related genes. In addition, to analyze whether the overexpression of *SiNRX1* in *Arabidopsis* would affect the expression of *AtNRXs*, *AtNRX1* and *AtNRX2* were selected to observe the changes at the transcription level (**Figure S4**). Under normal conditions, there was no significant difference in the expression levels of *AtNRX1* and *AtNRX2* in WT and the OE3 line, suggesting that overexpression of *SiNRX1* may have no affect the expression level of *AtNRXs* in *Arabidopsis*. However, under drought and salt stresses, the expression level of *AtNRX1* in OE3 line was significantly higher than that in WT. The expression level of *AtNRX2* in the OE3 line was lower than WT under drought stress, but higher than WT under salt stress.

## Discussion

Foxtail millet has become a research hotspot in recent years due to its small genome, strong stress resistance and high nutritional value. *TRX* family genes in rice, *Arabidopsis* and wheat have been confirmed to participate in the regulation of plant growth and development, environmental stresses and other biological processes, with very important biological significance (Nuruzzaman et al., 2012; Chibani et al., 2021; Bhurta et al., 2022). It is worth mentioning that, due to its unique redox regulatory activity, TRX has received extensive attention in the study of plant resistance. There are still few reports about foxtail millet TRX family members. The systematic identification of the SiTRX family in the full genome will assist us in excavating the gene resources for stress resistance in foxtail millet, thereby producing available gene resources for the breeding and genetic engineering of crops. In this study, 35 candidate SiTRX members were identified from the foxtail millet genome. Gene structure analysis showed that exon–introns located in the same branch of the TRX family were similar in distribution (**Figure 2B**). Plants with fewer introns have been shown to be better able to handle stress. *TaTRX* genes with fewer introns have been identified in wheat and can respond quickly to leaf rust pathogens (Bhurta et al., 2022). However, the function of introns in gene expression requires further verification. It is speculated that the difference between NRXs and other types may be the result of long-term evolution and adaptation to environmental changes through analysis of conserved motif. Promoters, as important regulatory regions upstream of genes, contain many *cis*-acting elements, which play very important roles in regulating gene responses to biotic stress, abiotic stresses and specific expression in plant tissues (Sheshadri et al., 2016). A large number of abscisic acid response elements (ABRE), stress response elements (MBS) and antioxidant stress elements (AREs) were found in the promoter regions of *SiTRX* family genes (**Figure S3**). These results suggest that SiTRXs may play important roles in plant stress responses and stress resistance.

Most studies have focused on the characterization of typical TRX functions, while less attention has been paid to atypical TRXs. With the deepening of research, the biological functions of atypical TRXs have gradually attracted the attention of researchers. The NRXs, as a class of atypical TRX members, contain multiple redox-active sites and are located in the nucleus, which may indicate that the NRXs have a stronger biological activity. In this study, three SiNRXs, SiTRX33 (Type I), SiTRX20 (Type II) and SiTRX25 (Type III), were identified according to the conserved domain, active site and subcellular localization. NRX was originally found in the nucleus of the mouse, so it was named “nucleoredoxin”. Studies have shown that NRX may be a redox regulator of the transcription factors (Kurooka et al., 1997). However, Funato et al. (2006) found that NRX was mainly located in the cytoplasm, and NRX appeared to be a specific suppressor of Wnt-*β*-catenin signaling. Marchal et al. (2014) describe a new TRX system, NTR/NRX, which is located in the nucleus and cytoplasmic compartment of *Arabidopsis*. It was found that the AtNRX1 of *Arabidopsis* has a dual localization—nucleus and cytoplasm. Li et al. (2016) found the GbNRX1 of island cotton is located in the exoplasm under stress. In this study, the localization analysis of the NRXs shows that their members are located in the nucleus, cytoplasm and membrane. The subcellular localization of NRX in different species may be different and may play different functions in different locations. Biotic and abiotic stresses have been reported to lead to cellular translocations of TRX enzymes or the entire TRX/NTR system, suggesting that these enzymes are recruited to specific sites for their signaling or protective functions (Mata-Pérez and Spoel, 2019), so it is speculated that the subcellular localization of NRX in different plants may be different or displaced. In addition, SiNRX1, SiNRX2 and SiNRX3 showed no self-activation activity in yeast, suggesting that NRX may not function as a transcription factor in plants, but as a functional protein regulating the redox state of biological targets *in vivo*. The analysis of *cis*-acting elements showed that the promoter regions of *SiNRX1*, *SiNRX2* and *SiNRX3* all contained abscisic acid response elements (ABRE), a low-temperature-response element (LTR), a hypoxia-specific-induction element (GC-motif) and other plant-hormone-response elements and stress-response elements. These results indicate that NRX might be involved in the anti-retroviral response, which was also confirmed by qRT-PCR (**Figure 3**). Therefore, we speculate that *SiNRX1* also may play a critical role in stress tolerance in plants. This hypothesis was verified by further functional identification of *SiNRX1* transgenic *Arabidopsis*. *SiNRX1* overexpression *Arabidopsis* significantly increased the growth potential and survival rate under drought or salt stress (**Figure 5**).

Plant responses to water and salt stresses have much similarity in most metabolic processes (Datta et al., 2012). Osmotic stress is a common adverse outcome of the response to drought and salt stresses. Proline accumulation is the first response of plants under osmotic stress (Grzesiak et al., 2013). It has been reported that proline, as a non-enzymatic antioxidant, may be involved in ROS signal transduction, and proline metabolism is related to the tolerance of plants to environmental stress. It has been proven that, the higher the content of proline, the stronger the stress resistance of plants (Szabados and Savouré, 2010; Liang et al., 2013). *P5CS1* is a key gene for proline accumulation, and its increased expression is induced by drought and salt stresses (Ben Rejeb et al., 2014). In this study, the proline content of overexpressed plants was highly up-regulated compared with WT under stresses (**Figures 6B**, **7B**). The *AtP5CS1* transcript level was strongly induced in the transgenic lines under drought and salt stresses (**Figure 8**). MDA is considered an indicator of oxidative damage at the cellular level (Sevanian and Ursini, 2000). The MDA content in the OE lines was much lower than that in the WT (**Figures 6A**, **7A**). Previous studies have demonstrated that the lower the MDA content, the higher the tolerance to abiotic stress (Zhu et al., 2021).

Drought and salt stresses also can lead to the excessive accumulation of ROS, especially O_2_·^-^ and H_2_O_2_, leading to severe oxidative damage in plants (Skopelitis et al., 2006; Wei et al., 2017). The plant’s antioxidant enzyme system (POD, SOD and CAT) protects cells from stress by maintaining ROS at normal levels (Yadav et al., 2019; Zhong et al., 2020; Ampofo and Ngadi, 2021). To determine whether *SiNRX1* modulates ROS accumulation, we tested the activities of several antioxidant enzymes (CAT, POD, SOD) and analyzed the expression levels of stress-related and antioxidant-related genes by qRT-PCR. They have previously been reported to be associated with the stress response (Sakuma et al., 2002; Narusaka et al., 2003; Li et al., 2020). The results showed that the activities of CAT, POD and SOD in *SiNRX1* overexpressed *Arabidopsis* were higher than those in WT under osmotic stress (**Figures 6**, **7**). Accordingly, several genes related to ROS-scavenging were strongly induced in overexpression lines (**Figure 8**). Li et al. (2016) proved that GbNRX1 is involved in regulating ROS homeostasis in response to pathogen invasion. Studies in *Arabidopsis* suggest that AtNRX1 plays an important role in plant immune signal transduction by maintaining CAT in a reduced state, thereby protecting its H_2_O_2_-detoxifying activity (Kneeshaw et al., 2017). *TaNRX1*-overexpressed wheat also displayed increased antioxidant enzyme activity under drought stress, which had a positive regulatory effect on transgenic wheat (Zhang et al., 2021). This was consistent with our experimental results. Moreover, *AtNRXs* was not affected under normal conditions, but *AtNRX1* of the OE line showed higher expression levels than WT after stress (**Figure S4**). It is speculated that *SiNRX1* could induce an increase in *AtNRX1* expression levels under abiotic stress. *AtNRX1*, as a redox protein, directly or indirectly regulates the antioxidant enzyme system, eliminates excessive reactive oxygen species and maintains the stable state of reactive oxygen species. As a result, *SiNRX1*-overexpressed *Arabidopsis* reduces the oxidative damage of plants and shows a better growth phenotype. However, further research is needed to elucidate the molecular mechanism of *SiNRX1*. In addition, the above studies indicated that SiNRX1 is located in the nucleus, cytoplasm and membrane, supposing that SiNRX1 also plays an important role in the nucleus. It may improve the tolerance of plants by regulating transcription factors related to stress tolerance. What NRX does in the nucleus may be the key to further research.

In summary, through the genome-wide analysis of foxtail millet, a total of 35 candidate TRX members were identified, which were divided into 13 groups according to their evolutionary relationships. In this study, the phylogenetic relationships, chromosome distributions, gene structures, conserved motifs and *cis*-elements of 35 SiTRX members were characterized. In addition, the expression pattern of *SiNRXs* under abiotic stresses was analyzed. Due to the special structure of SiNRX1 and its strong induction under stress, the stress resistance function of *SiNRX1* was identified through the overexpression of *SiNRX1* in the model plant *Arabidopsis*. These results show that *SiNRX1* was involved in the responses to drought and salt stresses, and results provide evidence for the relationship between *TRX* family genes and abiotic stresses in foxtail millet.

## Data availability statement

The datasets presented in this study can be found in online repositories. The names of the repository/repositories and accession number(s) can be found in the article/**Supplementary Material**.

## Author contributions

XZ and JX designed the study. SZ and YY performed the bioinformatics analysis. SZ, TS, and HZ collected samples and performed data analyses. MZ and NL conducted qRT-PCR experiment. TS and MY performed subcellular localization analysis. SZ wrote the manuscript. YNY, SG, CX, and YT revised the manuscript. All authors read and approved the final manuscript.

## Funding

This study was funded by the National Natural Science Foundation of China (32171992, 31671693), Research Program of Science and Technology at Universities of Inner Mongolia Autonomous Region (NJZZ22132), Central Guidance on Local Science and Technology Development Fund of Mongolia Autonomous Region (ZY20200089), Program for Young Talents of Science and Technology in Universities of Inner Mongolia Autonomous Region (NJYT-19-A25), Key Research and Development Project of Shaanxi Province (2019ZDLNY04-05), Innovation and Entrepreneurship Training program for college students of Northwest A&F University (S202110712347).

## Acknowledgments

We would like to thank Yi Ding and Jiaxin Ma for their assistance in sample collections and RNA extraction.

## Conflict of interest

The authors declare that the research was conducted in the absence of any commercial or financial relationships that could be construed as a potential conflict of interest.

## Publisher’s note

All claims expressed in this article are solely those of the authors and do not necessarily represent those of their affiliated organizations, or those of the publisher, the editors and the reviewers. Any product that may be evaluated in this article, or claim that may be made by its manufacturer, is not guaranteed or endorsed by the publisher.
