# Supplementary material for: Genome-wide identification of foxtail millet’s TRX family and a functional analysis of SiNRX1 in response to drought and salt stresses in transgenic Arabidopsis
Source: Front Plant Sci. 2022 Sep 26;13:946037. doi: 10.3389/fpls.2022.946037 (PMC9549295; doi:10.3389/fpls.2022.946037)
Supplement: Supplementary file 1 [file DataSheet_1.docx]

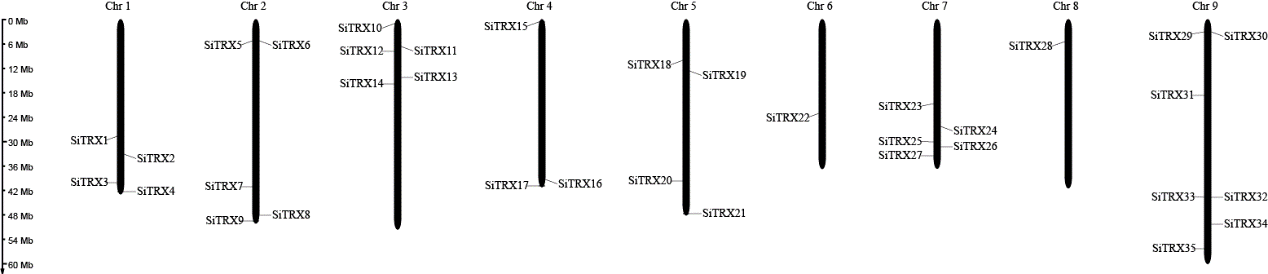


Figure S1 Chromosome distribution of *SiTRX* gene family members.


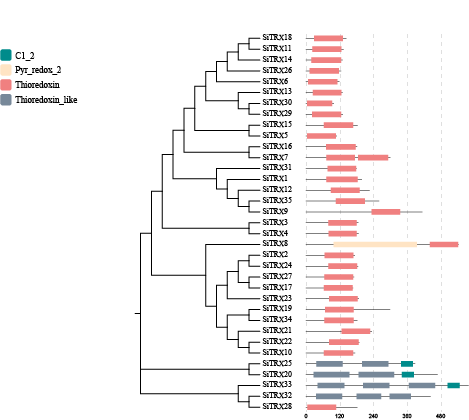


Figure S2 Domain analysis of SiTRX family. Domain indicated with boxes of different colors.


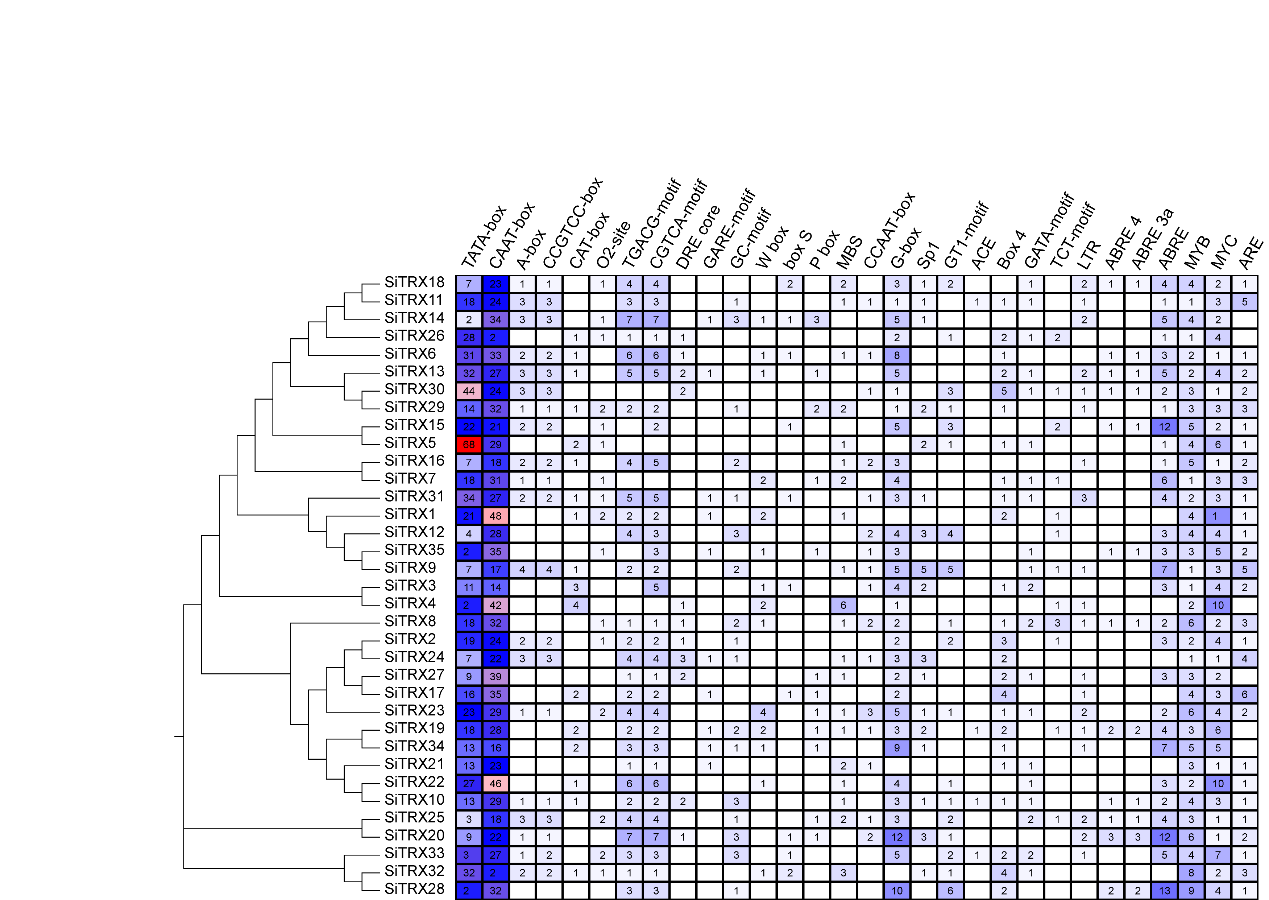


Figure S3. Cis-regulatory element prediction of SiTRX promoters. The sequences 2000 bp upstream of the ATG initiation codon of the SiTRX were obtained.


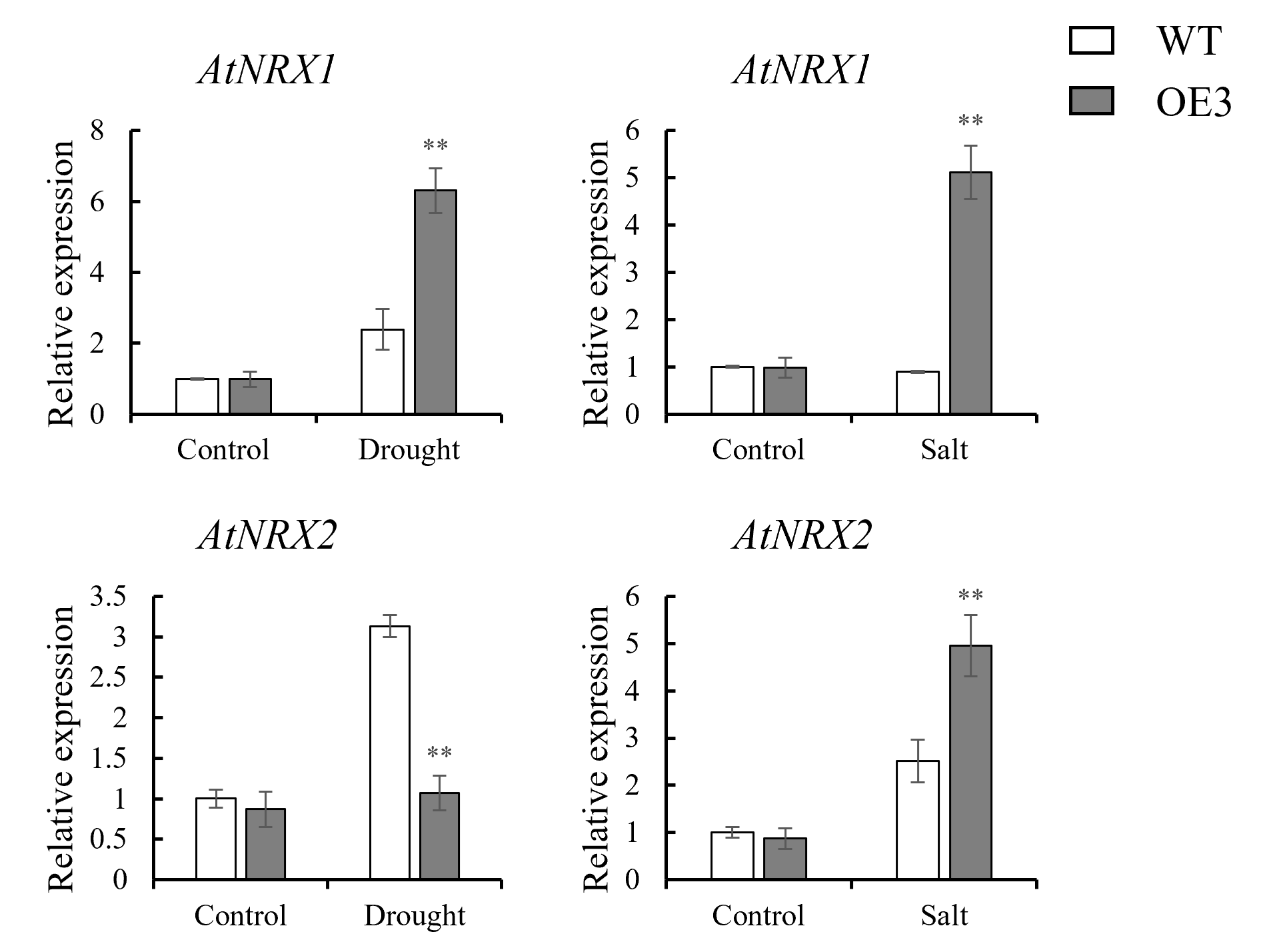


Figure S4. The expression levels of AtNRX1 and AtNRX2 in the OE3 line and WT under stresses. Stresses include drought stress and salt stress. Standard deviations are indicated by error bars (mean ± SD and n = 3). Student’s t-test were used to calculate significance: * indicates p < 0.05, and ** indicates p < 0.01.
